# Total Endoscopic Approach in Glomus Tympanicum Surgery

**Published:** 2017-11

**Authors:** Ahmad Daneshi, Alimohamad Asghari, Saleh Mohebbi, Mohammad Farhadi, Farhad Farahani, Mohammad Mohseni

**Affiliations:** 1 *ENT Head and Neck Research Center and Department, Iran University of Medical Science, Tehran, Iran.*; 2 *Skull Base Research Center, Iran University of Medical Science, Tehran, Iran.*; 3 *Department of Otolaryngology, Hamedan University of Medical Science, Hamedan, Iran.*

**Keywords:** Endoscope, Glomus tympanicum, Surgery

## Abstract

**Introduction:**

Glomus tympanicum (GT) is a benign primary tumor of the middle ear. The evolution of endoscopic ear surgery has allowed for an alternative approach to managing this vascular tumor. The purpose of this study was to evaluate an endoscopic approach in GT surgery, and also to investigate its applicability and feasibility.

**Materials and Methods:**

Prospectively, 13 class I and II patients, according to the Glasscock-Jackson glomus classification, were candidates for management via a transcanal endoscopic approach. Patients were categorized into three groups according to the location of the tumor in the middle ear. Group A consisted of patients with tumors located anteriorly while occupying the Eustachian tube. Group B were patients with tumors located on the promontory with entirely visible tumor borders. Patients in Group C had tumors that occupied the entire middle ear. Under specially designed flap elevation and hemostasis, the tumors were completely removed using an endoscopic technique.

**Results:**

Based on the classification criteria, three patients fell into Group A (30%), six into Group B (46%), and three into Group C (23%). The principal chief complaint was pulsatile tinnitus that disappeared after surgery in most cases. Hearing status was mostly mixed hearing loss. No change was detected in bone conduction after surgery, but air conduction was improved in nine cases. No major complication or recurrence was observed over 30 months of follow up.

**Conclusion:**

Improved exposure and access in the endoscopic transcanal approach to GT leads to safe, rapid, and reliable tumor removal, as well as allowing comfortable surgery for both the surgeon and most patients.

## Introduction

Glomus tympanicum (GT) is a benign primary tumor derived from the paraganglionic tissues of the middle ear (1). The main treatment modality for GT tumors is surgical resection (2). Surgical approaches are mostly transcanal, but depending on the size and extension of the tumor, transmastoid/facial recess and canal wall down mastoidectomy are sometimes required (3). Despite the long history of endoscope use in ear surgery, owing to the size of the external canal and the bony boundaries of the middle ear and instrument, this technique is not very popular. Endoscopic ear surgery for different pathologies, including stapes surgery (4,5), middle ear surgery (6,7), and tympanoplasty (8), was reintroduced to otolaryngologists in the last decade and is more popular these days. With the help of an endoscope, the need for external incision, excessive soft tissue dissection, and mastoidectomy can be avoided, and instruments only have to pass through the external ear canal.

The purpose of this study was to evaluate use of an endoscopic approach in GT surgery and to investigate its applicability and feasibility. In this article, a total endoscopic approach to selected cases of GT is described.

## Materials and Methods

**Fig 1:**
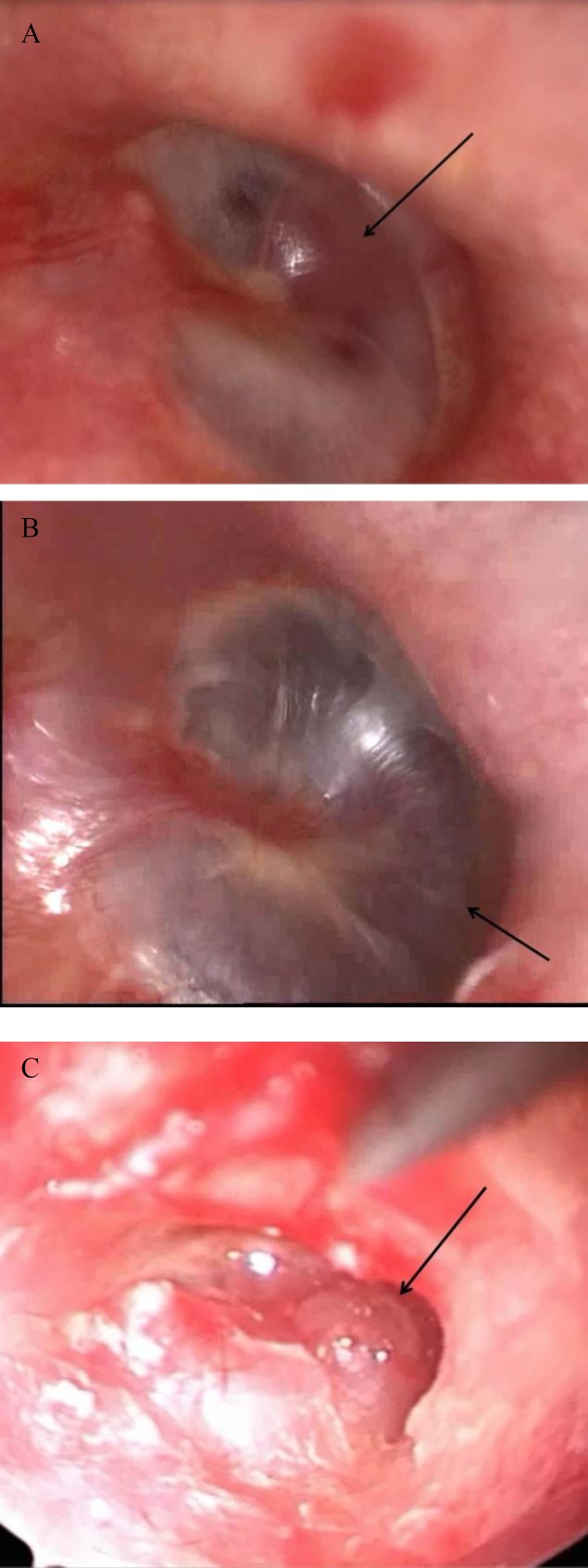
Endoscopic view, Group A, B and C.

**Fig 2:**
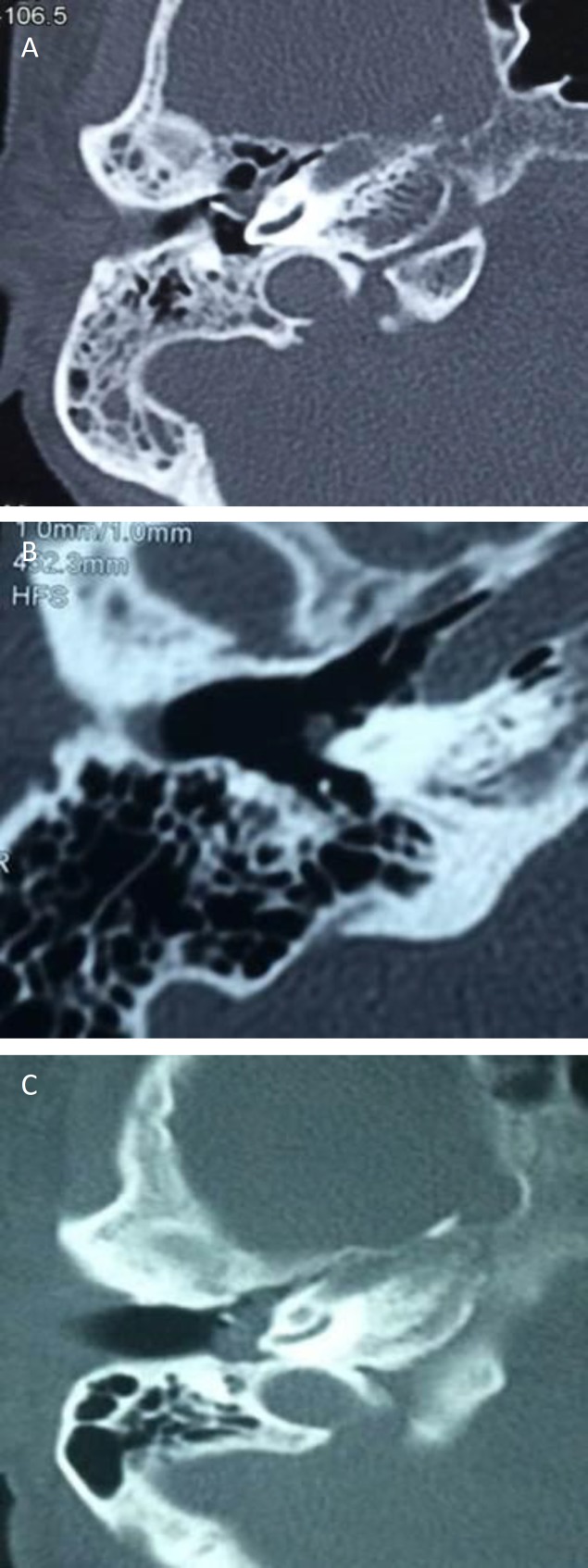
Imaging of subgroups, Group A (a), Group B (b), Group C (c)

Between January 2013 and January 2016, diagnosed cases of GT tumor were selected for endoscopic management. Patient demographic data and preoperational evaluation, including tympanometry, audiometry and imaging (computed tomography [CT] and magnetic resonance imaging [MRI]) were recorded. According to the Glasscock-Jackson glomus classification, class I and II patients were candidates for management through an endoscopic transcanal approach (9). Patients were categorized into three subgroups according to the position of the tumor (Table.1, Fig.1). Sample CT images of the three categories are shown in Figure 2.

**Table 1:** Glasscock-Jackson classification system

Grade Definition
1. Tumor margins completely visible on otoscopy
2. Tumor filling the middle ear
3. Tumor filling the middle ear and in the mastoid
4. Tumor extending through the tympanic membrane into the external auditory canal

Thirteen patients with a GT diagnosis were included in the study, and their data were recorded (Table.2). The principal chief complaint was pulsatile tinnitus, and the examination showed a red mass in the middle ear behind the tympanic membrane (TM) in most cases.

**Table 2:** Categorization of patients according to tumor location in the middle ear

**Group A**	**Group B**	**Group C**
Tumor located anteriorly and occupying Eustachian tube(ET) opening	Tumor located on the promontory. Entire tumor boarder visible	Tumor fully occupied the middle ear and border not visible through the TM

*Procedure*

The procedure was performed under general anesthesia with patients in the supine position, with head extension at about 30º and turned 45º toward the opposite side of the involved ear. The video equipment was placed in front of the surgeon and the instrument trolley and scrub nurse were positioned at the head. Endoscopes 4-mm in diameter and 18-cm in length, with angulation of 0º and 30º were used. A high-definition monitor (Karl Storz, Tuttlingen, Germany) and camera with an AIDA^TM^ storage system were used. After Prep and Drape and canal irrigation, lidocaine 2% and adrenaline (1/100,000 dilution) was injected under the planned tympanomeatal flap to aid the elevation and also to reduce bleeding. The endoscope was held in the left hand while the instruments were used with the right hand. An extra hand was typically actively involved in intermittent irrigation and suctioning. An angled lens was used to identify the border and to look for remnants in corners.

**Fig 3:**
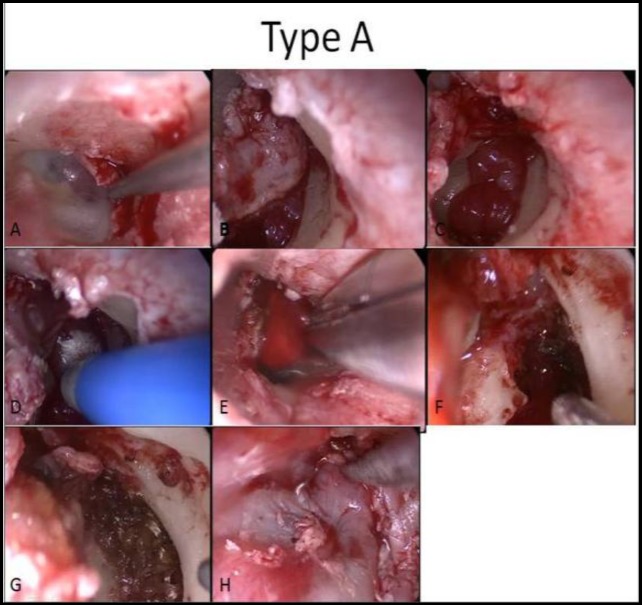
Type A, Endoscopic view, flap elevation, hemostasis and tumor removal (A–H)

**Fig 4:**
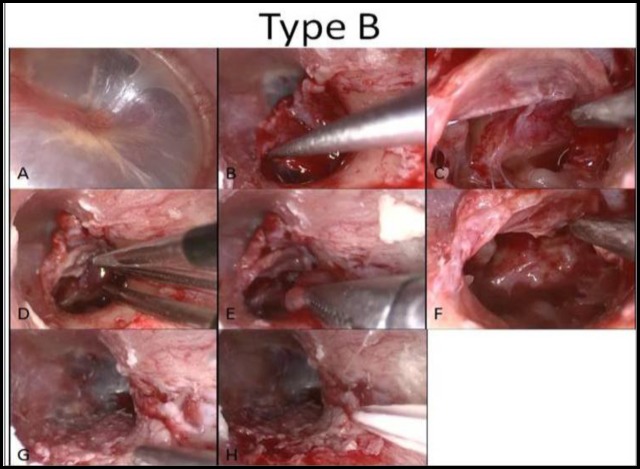
Type B, Endoscopic view, flap elevation, hemostasis and tumor removal (A–H)

**Fig 5:**
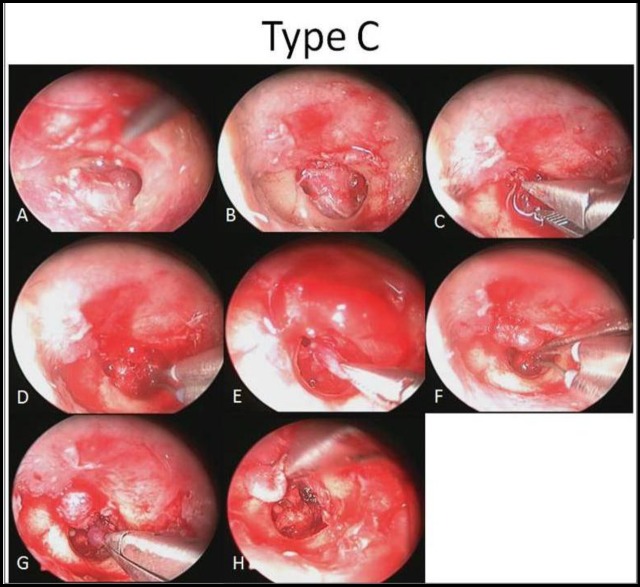
Type B, Endoscopic view, flap elevation, hemostasis and tumor removal (A–H)

For Group A, an anterior-based tympano- meatal flap (from 12 to 6 o’clock) was designed 6 mm away from the TM, and elevated with a round and blaster knife (Fig.3). The middle ear and tumor was exposed, the tumor was coagulated (using Stammberger bipolar suction or an argon plasma coagulation probe), and completely removed without ossicular manipulation. The ET opening was checked to ensure that there was no presence of the tumor. For Group B, the tympanomeatal flap design commenced from the 3 to 9 o’clock position, and the tumor was removed after coagulation in the same way (Fig.4). For Group C, the flap was designed from 2 to 10 o’clock using the same method, and the tumor was completely removed. Appropriate care was needed to preserve the ossicular chain continuity (Fig. 5). After tumor removal and hemostasis, the tympanomeatal flap was returned back in position, and 1-ml tissue adhesive glue (Glubran Tiss; GEM, Italy) was applied over the incision line without external auditory canal packing or dressing.

## Results

A prospective chart review, patient interviews, and examination were used for data collection. Glomus tumors were confirmed histopatholo- gically. Nine cases were female and four cases were male, with a mean age of 54 years. According to the Glasscock-Jackson classification, six cases were classified as class I (46%) and seven as class II (54%). 

Four cases fell into Group A (30%), six into Group B (46%), and three into Group C (23%) (Table.3). In seven cases, the tumor was on the right side (54%) and in the other six cases, the left side was involved (46%). There were no hormonal-related symptoms.

**Table 3:** Demographic data and tumor classification

**Age**	**Sex**	**Tumor side**	**Tumor class**	**Subgroup**
47	F	Lt	II	B
44	F	Lt	I	A
50	F	Rt	II	B
44	F	Lt	I	A
66	F	Rt	II	B
68	F	Rt	II	B
68	F	Lt	I	B
60	M	Lt	I	A
62	M	Rt	I	B
52	M	Rt	I	A
47	F	Rt	II	C
49	F	Rt	II	C
46	M	Lt	II	C

The principal complaint in most patients was pulsatile tinnitus, which disappeared after surgery, except in one patient (#7) where the tinnitus was not of the pulsatile type. Hearing was evaluated according to the American National Standards Institute (ANSI) guidelines (ANSI S3.21-1978; revised 1997), in which a loss of 0–25 dB indicated normal hearing, 26 to 40 dB indicated mild, 41 to 55 dB indicated moderate, 56 to 70 dB indicated moderately severe, and 71 to 90 dB indicated severe hearing loss, while >90 dB indicated profound hearing loss or deafness (10). The most common patterns of hearing loss were sensorineural hearing loss (SNHL; four cases), mixed (four cases), conductive hearing loss (CHL; three cases), no hearing loss (one case). One of the cases, whose tumor was an incidental finding, was previously deaf (#9), and was therefore excluded from the hearing analysis. One case had normal hearing (#3). In the postoperative state, most cases experienced improvement in their hearing. No change was detected in bone conduction, but air conduction was improved in nine cases, although two cases still had mild air-bone gap (Table. 4).

**Table 4:** Conductive hearing status (air conduction) before and after surgery.

Hearing status*	**Number of cases (Preoperative) **	**Number of cases (Postoperative)**
0–25 normal hearing	4	10
26 to 40 dB (mild hearing loss)	7	2
41 to 55 dB (moderate)	1	-
56 to 70dB (moderately severe)	-	-
71 to 90 dB (severe)	-	-
> 90 dB (profound or deaf)	1	1

* According to American National Standards Institute Guideline (ANSI)

None of the cases received embolization. In four cases (#3,4,5, and 6), argon plasma coagulation was used before tumor manipulation for the purpose of shrinkage and hemostasis. Fine bipolar cautery (Stammberger bipolar suction cautery) was used in the other cases. The mean duration of the surgery was 1 h, ranging from 45 min to 2 h. The maximum duration was in a patient from Group C (#11), in which case Surgicel was needed for hemostasis. The average bleeding volume was 100 ml; however maximum bleeding was less than 150 ml.

In type-C cases, TM perforation was inevitable. For its reconstruction, tympanoplasty was performed with cartilage. In six cases (#1,4,5,6,7, and 8), the middle ear was packed with gelfoam, while in case 11 it was packed with gelfoam and Surgicel. In the other cases, tympanomeatal flaps were repositioned without middle-ear packing. The external ear was left free of packing or gelfoam only in Group C. For the other cases, the tympanomeatal flap was put back in position and secured using tissue adhesive glue. Six cases were discharged on the day of surgery, and others were discharged on the next day.

Patients were visited at least every 2 or 3 months in a scheduled manner. Mean follow up was 20 months, and some patients were followed up for more than 3 years. All patients were assessed for any complications, morbidity, or recurrence. No adverse effect or complication was noted, and there was no recurrence or residual growth. In the most recent visit, all patients were content and had no significant complaints.

## Discussion

This study is a prospective review of 13 selected cases of GT treated endoscopically over 3 years in a tertiary referral center. The goal was to evaluate the role of endoscopy and its applicability for the treatment of GT, and also to investigate complications, morbidity, and hearing status of patients following therapy. Thirteen cases were investigated and all demographic and imaging data were collected.

The patients’ ages ranged from 41 to 68 years, with a mean of 54 years. There were nine female patients and four male patients, which is a similar ratio to previous studies (3), but differs from a report by Carlson et al., which showed a 9/1 female-to-male ratio (11).

Many GT tumors can be accessed through the ear canal. According to the class of tumor, sometimes a retroauricular or transmastoid approach is needed (12). In 115 cases reported by Carlson et al., 32% of tumors were removed via the transcanal route. However, in this study, 26 cases were stage III and IV according to the Glasscock-Jackson classification (11). The endoscopic ear technique was introduced years ago and was popularized and promoted by Tarabichi, Thomassin , Daneshi, and Marcholini for different ear surgeries and skull-base approaches (5,13-15). There were no operative mortalities, complete gross surgical removal was achieved in all cases, and facial nerve function remained intact. Three TM perforations occurred which were repaired in the same setting. Rohith et al. reported 12% TM perforation and 12% canal stenosis in 17 cases (12).

**Fig 6:**
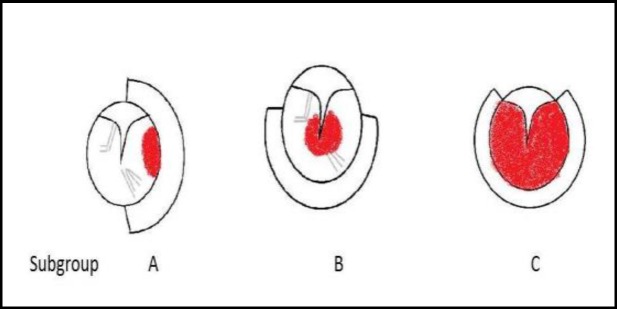
Tympanomeatal flap design in the subgroups

There was no significant bleeding. The average and maximum bleeding was 100 ml and 150 ml, respectively. Other studies also reported a lack of significant bleeding, except for O’Leary et al. who reported significant intraoperative bleeding in their series (16). Bipolar or laser cauterization is very helpful to reduce the volume of bleeding. Durvasula et al. described their tumor resection using a KTP or diode laser without complication or recurrence (17).Tumor location and stage modifies the flap design and categorization. Different tympanomeatal flaps are designed for better access and exposure. Balkany et al. described different tympanomeatal flaps to access tympanomastoid pathologies (18). In this study, these flaps were modified to access the lesion, as shown in (Fig. 6).

Hearing evaluation was performed before and after surgery using pure tone audiometric studies. Normal hearing is defined as less than 25 dB loss and 88% speech discrimination (3). Hearing was not worsened, but air conduction was improved to normal in 10 patients because of tumor removal and improved middle-ear function following surgery. The non-disruptive technique, avoiding damage to the ossicles, allowed for the preservation of hearing. Cartilage tympanoplasty was performed simultaneously in some injured cases. This point explains the remaining mild air-bone gap after surgery in case numbers 11 and 13 within the follow-up period (Table.2). Papaspyrou et al. showed an improved hearing threshold and decrease in air-bone gap at most frequencies, particularly 3,000 kHz, after surgery (19).

Because use of a packing technique is avoided, the patients felt more comfortable immediately after surgery. In the three cases only that needed tympanoplasty, the external auditory canal was packed with gelfoam.

The recurrence rate is quite low but may occur at any time, so life-long follow up is recommended (3). Sanna et al. reported one case of recurrence in their series (68 cases) after 9 years (20). A review over 4 decades in the Vanderbilt University Center, Nashville reported no recurrence in cases with total gross removal (11). The average follow up for patients was 25 months, with no patients showing recurrence during that time. Follow up was scheduled on a routine basis and endoscopic or microscopic ear examination and CT or MRI imaging were performed when required.

Endoscopy is thoroughly applicable and very useful in exposing corners and hidden areas that might not be visible within the direct view of a microscope. One-handed surgery seems a limitation to this technique, which could be conquered over time by becoming familiar with the device and technique. Endoscopic ear surgery has a learning curve and should be initiated with simpler cases. This learning curve provided us with easier and safer operations in our latest cases. The use of two or three hands is possible when two expert otologist surgeons work simultaneously. Because of the absence of incision or canal packing, patients were very satisfied with the procedure, and returned to normal life rapidly.

## Conclusion

An endoscopic transcanal approach to GT is feasible. This method provides better exposure and access, therefore leading to safe, rapid, reliable tumor removal without sutures, or canaloplasty, and is a comfortable surgery for most patients.
